# Hazard Assessment of Antioxidants as Contaminants of Concern

**DOI:** 10.1021/acs.estlett.5c01217

**Published:** 2026-03-02

**Authors:** Carolin Seller-Brison, Franziska Weissbach, Kjell Jorner, Martin Scheringer, Kathrin Fenner

**Affiliations:** † 30964 University of Zurich, Department of Chemistry, Winterthurerstrasse 190, 8057 Zurich, Switzerland; ‡ Eawag, Department of Environmental Chemistry, Überlandstrasse 133, 8600 Dübendorf, Switzerland; § 27219 ETH Zurich, Department of Chemistry and Applied Biosciences, Institute of Chemical and Bioengineering, Vladimir-Prelog-Weg 1-5/10, 8093 Zurich, Switzerland; ∥ ETH Zürich, Department of Environmental Systems Science, Universitätstrasse 168092 Zürich, Switzerland

**Keywords:** Synthetic antioxidants, natural antioxidants, primary antioxidants, secondary antioxidants, hazard assessment, transformation products

## Abstract

Antioxidants (AOs) are increasingly detected in the environment, in aquatic organisms, and in human biosamples. Therefore, we performed a comprehensive hazard assessment of more than 500 natural and synthetic AOs by exploring their recalcitrance toward mineralization (not readily biodegradable, NRB), persistence (P), bioaccumulation (B), toxicity (T), mobility (M), and formation of toxic transformation products (TPs). Literature experimental data complemented with *in silico* predictions revealed that 60% of the AOs classify as NRB and T, out of which 31.6% classify as P and T. Of highest concern according to EU regulations are PBT or PMT compounds, with 4.8% and 2.1% of the AOs studied, respectively, falling into these categories. All AOs classified as PBT or PMT are of synthetic origin and, by majority, primary AOs, i.e., phenolic or amine compounds. Further, most predicted toxic TPs stem from phenolic and amine structures. Natural or nature-identical primary AOs or secondary AOs, *e.g.,* ester or organic sulfur compounds, are less often classified as hazardous. Only 19 AOs were identified as not fulfilling any of the hazard criteria, highlighting the need for more experimental data on AO hazards for regulatory purposes, as well as for further research on safe AO alternatives.

## Graphical abstract

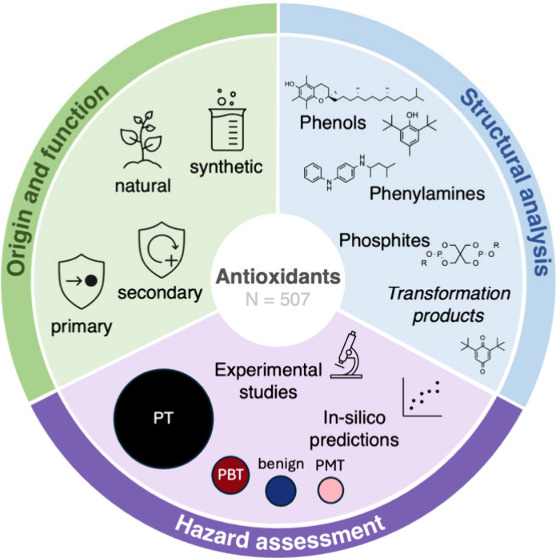

## Introduction

Antioxidants (AOs) are chemicals that slow down hydrocarbon oxidation, which is why they are used extensively to protect materials and products like polymers, rubber, fuels, pharmaceuticals, or foodstuffs from oxidative damage. Chemicals used as AOs have different mechanisms of action. Some AOs prevent formation of reactive oxygen species by absorbing light, dissipating it as thermal energy and preventing it from initiating oxidation.^1^ Others, referred to as chain-breaking AOs, stop autoxidation of materials by being oxidized faster than the material they protect.^2,3^ Chain-breaking AOs can be further classified by structures and function. Primary AOs, *e.g*., phenols and amines, react rapidly with free radicals. Secondary AOs, *e.g*., phosphites and organic sulfur compounds, delay oxidation by chelating metal ions or decomposing hydroperoxides and are mostly applied synergistically with primary AOs. Various secondary AO structures can act as primary AOs as well, although their main role remains to decompose hydroperoxides.^3^

Due to their widespread application, AOs have been detected in various environmental compartments, *e.g*., in ground- and surface water in the ng L^–1^ range,^4,5^ in sediment and soil at ng g^–1^ to μg g^–1^ concentrations,^6,7^ as well as in outdoor aerosol particles and indoor dust.^8,9^ Biomonitoring studies found AO traces in aquatic species,^10^ and in human urine or blood.^11,12^ Widespread exposure to AOs is problematic as some AOs are persistent and bioaccumulative,^13–15^ while others may form toxic transformation products (TPs).^16^ For example, the rubber antioxidant 6PPD (*N* ^1^-(4-methylpentan-2-yl)-*N* ^4^-phenylbenzene-1,4-diamine) and its quinone TP (6PPD-Q), both found in human urine and blood samples, can cause adverse effects such as neurotoxicity or reproductive toxicity.^11^ Further, 6PPD-Q is acutely toxic to aquatic life and has caused massive coho salmon mortality events.^16^ Hence, from a safety perspective, it is not sufficient to only study the AO parent compound – unless fully mineralized, AOs must also be investigated for toxic TPs formed during degradation.^12,33^

As of today, some AOs, such as the primary AO BHA (butylated hydroxyanisole) or the UV absorber UV328 (2-(2H-benzotriazol-2-yl)-4,6-di-*tert*-pentylphenol), have already been restricted for certain uses or banned completely.^15,17^ Yet, little is known about impacts on environmental and human health for the majority of chemicals used as AOs. This highlights a need to systematically identify and characterize less studied AOs, and points to the importance of finding smart substitutions.

Against this background, the aim of this study is to identify a broad range of organic chemicals used as AOs and to gain an overview of their hazards, *i.e*., (i) recalcitrance to mineralization, (ii) persistence, (iii) toxicity toward humans and aquatic life, (iv) potential to bioaccumulate, and (v) mobility within natural or engineered systems. We first collected data from regulatory databases and the scientific literature to explore hazard properties of well-studied AOs, as well as to highlight existing data gaps. Second, we employed state-of-the-art *in silico* tools to fill data gaps and allow for a full assessment of hazards. Third, we identified potential TPs and evaluated their aquatic toxicity. Herewith, this study provides a hazard assessment of a wide range of AOs, systematically elucidates AOs of high concern, and highlights trajectories toward structures with more favorable safety profiles. It complements recent large-scale assessments of plastic chemicals, which are based exclusively on hazard classifications from regulatory authorities and industry self-declarations,^18,19^ with a more fine-grained analysis for AOs specifically.

## Materials and Methods

### Selection and Structural Grouping of AOs

We curated a list of organic chemicals used as AOs based on specific peer-reviewed literature on polymer additives, primary and secondary AOs, and natural AOs.^5,9,17,19–27^ As the most common AO structures contain a phenolic group, we further complemented our list with a systematic search of the Web of Science Core Collection for phenolic AOs as detailed in SI 1.1–1.3.^28,29^

### Hazard Assessment of AO Parent Compounds

We determined the selected AO parent compounds’ properties, *i.e*., recalcitrance to mineralization (not readily biodegradable, NRB), persistence (P), toxicity toward humans and aquatic life (T), potential to bioaccumulate (B), and mobility within natural or engineered systems (M). To perform the hazard assessment, we employed cutoff criteria for the respective properties as defined in different EU regulatory frameworks (Table 1), but there are similar approaches in other parts of the world, such as the Canadian system under CEPA.^30^ Data were collected from the ECHA database (November 2024, experimental and predicted data),^31^ the USEPA ECOTOX database (November 2024, experimental data),^32^ the substances’ safety data sheets provided by vendors (experimental data, Appendix T5), PubChem (experimental and predicted data),^33^ the International Agency for Research on Cancer Classified Agents List (experimental data),^34^ the Japanese GHS classifications list (experimental data),^35^ and the peer-reviewed literature.^19,36^ Data gaps were filled using OPERA (*K*_ow_, *K*_oc_, bioconcentration factor (BCF)),^37^ EpiSuite v 4.11 (NRB, P, aquatic T),^38^ Toxtree 3.1.0 (Cramer classes),^39^ and the RB prediction workflow based on Körner et al.^40^ (https://biodegradability-prediction-app.streamlit.app/). In evaluating an AO’s hazards, priority was always given to experimental data.

**Table 1:** Hazards Considered and Cut-off Criteria Applied to Separate between Hazardous AOs and AOs for Which There Is No Evidence of Hazard^a^

Hazard	Hazard cutoff criteria
NRB	OECD 301 study outcomes: <70% removal of dissolved organic carbon^41^
	BIOWIN6 predictions: AO does not biodegrade fast (probability <0.5),^42^ Körner et al.^40^ prediction outcomes: NRB
	
P	OECD 308/309 simulation study outcomes: half-lives (DT_50_) >40 days in freshwater or >120 days in sediment^42,43^
	BIOWIN3 predictions: ultimate biodegradation time frame prediction ≥months (value <2.2)^42^
	
**T**	**Toxicity toward aquatic life**
	Acute toxicity: EC_50_ (48 h) for aquatic invertebrates or LC_50_ (4 days) for fish is <1 mg L^–1^ ^44^
	Chronic toxicity: NOEC or EC_10_ for marine or freshwater organisms is <0.01 mg L^–1^ ^43,44^
	log *K*_ow_ > 4 if acute and chronic toxicity cannot be assessed otherwise^44^
	AO safety data sheet suggests acute and chronic toxicity to aquatic life
	Toxicity toward humans
	CMR compounds: all AOs that meet the criteria for classification as carcinogenic (C, category 1A or 1B), germ cell mutagenic (M, category 1A or 1B), toxic for reproduction (R, category 1A, 1B, or 2), or specific target organ toxicity after repeated exposure (STOT RE, category 1 or 2)^43^
	ED compounds: all AOs that meet the criteria for classification as endocrine disruptor (ED)^43^
	AO safety data sheet suggests liver toxicity, or neurotoxicity
	Toxtree predictions: Cramer Class III^39^
	
B	Bioconcentration factor (BCF) in aquatic species >2000 ^43^
	
M	log *K*_oc_ is <3 ^43^

a Regulatory guidelines used to derive the applied cut-off criteria are referenced accordingly. Further details are provided in Table S1. When more than one cut-off criterion is listed for a respective hazard, priority was given to them in descending order.

### Structural Grouping of AOs

We used SMARTS pattern matching to identify structural features that frequently occur in the collected set of AOs and to create the following, partially overlapping subclasses: phenols, amines, quinones, phosphorus-containing structures (phosphates and phosphites), aliphatic alcohols, carboxylic acids, esters, sulfur-containing structures, and structures containing tin. We computed Morgan Fingerprints^45^ of size 2048 and radius 2 for all structures, reduced their dimensionality to 50 using principal component analysis (PCA), and then applied t-distributed stochastic neighbor embedding (t-SNE)^46^ to visualize them in two dimensions. The perplexity hyperparameter for the t-SNE embedding was chosen visually to a value of 30 and the distance metric used was euclidean.

### Toxicity of Transformation Products

Our literature review yielded information about TPs of six AOs, *i.e.*, BHT (butylated hydroxytoluene), BHA, 6PPD, IPPD (*N*-isopropyl-N′-phenyl-1,4-phenylenediamine), AO168 (tris­(2,4-di-*tert*-butylphenyl) phosphite) and TBHQ (*tert*-butylhydroquinone). We used enviPath^47^ to determine potential TPs of the remaining 501 AOs. We limited predictions to 30 TPs per AO, a number which had previously been shown to provide good coverage of experimentally observed TPs while optimizing specificity.^48^ TPs with an assigned probability ≥ 50%, and hence increased likelihood for exposure, were assessed for their potential aquatic toxicity using ECOSAR predictions available through EpiSuite v 4.11^38^.

## Results and Discussion

### Data Availability

Our literature search and data curation steps resulted in a list of 507 AOs and 9 TPs, *i.e*., 186 phenols, 104 amines, 51 phosphor-based structures, and others (Appendix T1). Of those, 103 are natural or nature-identical AOs, while the remaining 413 AOs are synthetic compounds (Appendix T3). The availability of literature experimental data describing the AOs’ hazards is shown in Figure 1A, Figure S4, and Appendix T2. Most experimental information was found for T and NRB with data for 74% and 44% of the selected AOs, respectively. Considerable data gaps exist for P, B and M with data only available for 0.05%, 15.7%, and 29.5% of the AOs, respectively.

**Figure 1:**
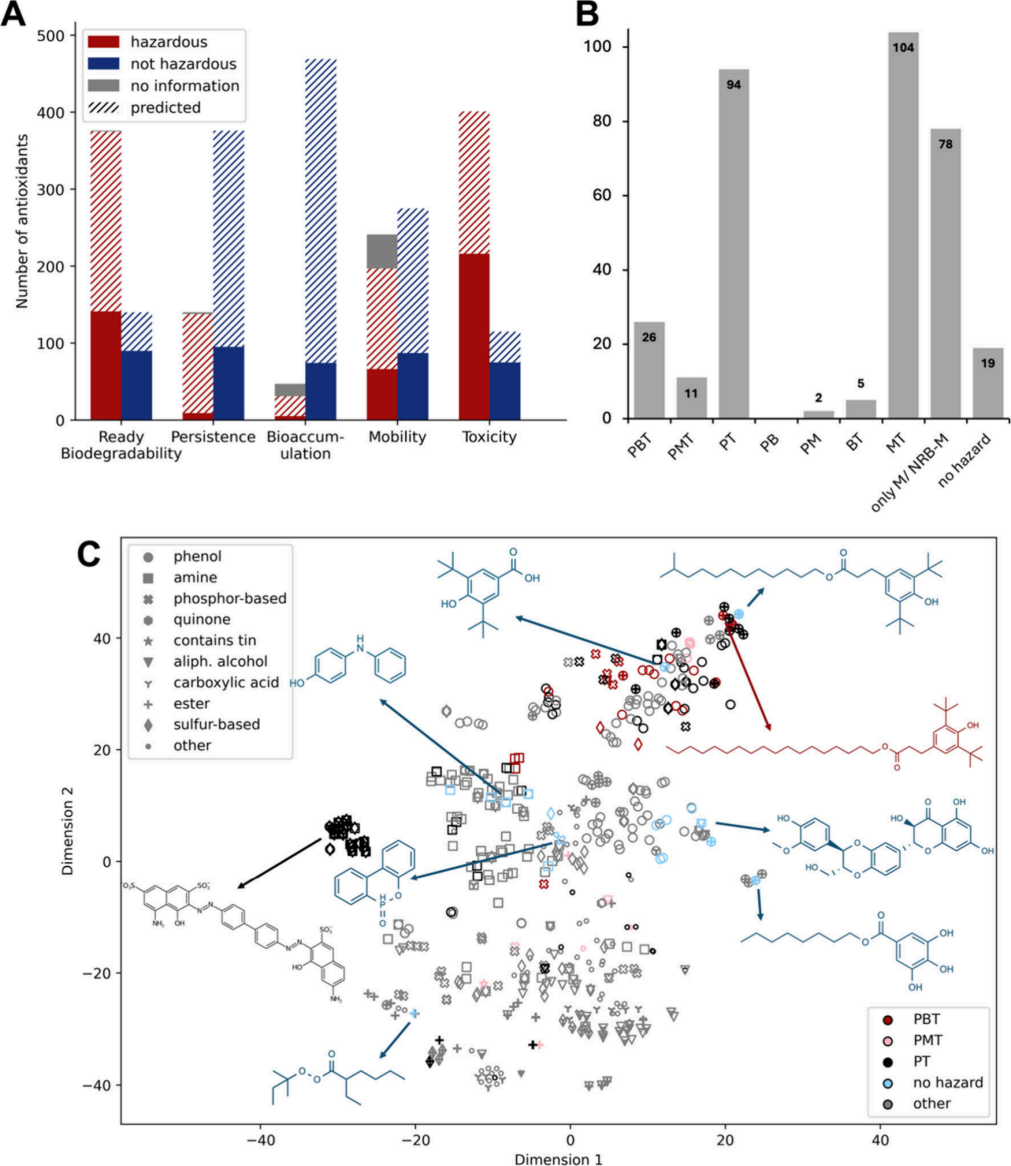
(A) Data availability and *in silico* predictions of biodegradability, persistence, bioaccumulation, mobility, and toxicity. Solid bars represent compounds with publicly available hazard data, hatched bars represent compounds for which we estimated their hazards. (B) Grouping of hazardous AOs based on data shown in Figure 1A. (C) t-SNE plot showing clustering of chemicals based on structural fingerprints and hazards (indicated by colors). Frequently occurring functional groups are indicated by markers. Single compounds may contain multiple functional groups and are plotted with overlapping markers.

### Model Predictions of AO Hazards

To fill the considerable data gaps, the use of *in silico* tools was necessary to allow for a full hazard assessment of all AOs (Figure 1A). We compared prediction and literature experimental data for as many substances as possible. For example, for NRB, predictions from BIOWIN6 ^38^ and the prediction tool of Körner et al.^40^ aligned with the experimental results in 76 and 77% of the cases, respectively (Figure S1, literature experimental data n = 231). For aquatic toxicity, only half of the ECOSAR^38^ predictions for acute toxicity toward invertebrates and fish were within the same order of magnitude as the experimental values. Yet, when compared to the T cutoff criterion (*i.e*., 1 mg L^–1^, Table 1), these discrepancies nevertheless resulted in the same T classification for 80% of the AOs (Figures S2 and S3).

Discrepancies between literature experimental data and *in silico* predictions prompted us to investigate BIOWIN^38^ predictions more closely. As a group contribution method, BIOWIN^38^ facilitates interpretation of estimates based on individual structural contributions. The most influential structural features were found to be the following: ester groups, aromatic alcohols and linear carbon chains affect mineralization positively, while high molecular weight is penalized. In case of AOs, this approach led to false hazard classifications, *e.g.,* for phosphite structures, which are estimated to be persistent, even though previous studies have shown that it is mostly their phosphate TPs that reach the environment, suggesting rather fast transformation of the parent phosphites.^22,49^ However, BIOWIN^38^ does not recognize any phosphite fragments and only considers the rather large molecular weight of these molecules (Appendix T1), falsely suggesting persistence of parent phosphites. While this example shows that *in silico* predictions can introduce systematic errors into hazard classification of AOs, they are needed as long as experimental data is lacking, reflecting a common issue in regulatory hazard assessment of industrial chemicals.^50^

In terms of toxicity assessment, both experimental and *in silico* toxicity determination is especially challenging for AOs due to their reactivity. For AOs that autoxidize or hydrolyze during the time frame of a given experiment, experimental toxicity data is uncertain and likely represents an unknown and changing mixture of parent AO and TPs.^51^ If an AO is stable but exhibits a specific mode of toxic action, prediction tools tend to underestimate toxicity.^52^ These prominent limitations highlight the great need for further research into the hazards of AOs.

### Hazard Assessment

If only AOs are considered for which there are experimental data available, 61% of these AOs are NRB within the time frame given in the OECD 301 guideline and 74.7% are T (see solid bars in Figure 1A, Figure S4). A detailed splitting up of the toxicity data into acute and chronic toxicity to aquatic life, human toxicity and specific toxicities, *i.e*., CMR and ED, is given in Figure S4 and Appendix T2. Data from higher-tier persistence studies are available only for 28 out of the 516 selected AOs. According to those, 18 AOs are P (Appendix T2). The data available for B suggest that AOs rarely bioaccumulate. Filling data gaps with *in silico* predictions and analyzing across all 516 AOs confirmed the hazard trends observed in the set of AOs with experimental data, *i.e*., that NRB and T are the most prominent hazards of AOs with 72.8% classified as NRB and 77.7% as T. Further, across all data, 41.7% of the AOs are classified as M, 26.5% as P (corresponding to 36.5% of those classified as NRB), and 6.2% as B (Figure 1A). As elaborated above, these classifications bear some uncertainty due to limitations of *in silico* hazard predictions. Yet, the data set still allows to observe overall trends regarding potential hazards of AOs.

We used all experimental and predicted data to identify AOs that combine two or three hazard properties (Figure 1B) to identify compounds of higher concern from a safety perspective. Most notably, 60% of the AOs classified as both NRB and T, 31.6% of which are also P and T. Further, 4.8% and 2.1% of the AOs were classified as PBT or PMT substances, respectively, and would thus qualify as substance of high concern according to EU regulations, warranting further exploration of use scenarios, exposure levels, and environmental concentrations.^43^ In fact, three of our identified PBT substances have already undergone a full regulatory hazard and risk assessment and have been classified as Substances of Very High Concern (SVHCs) under REACH, *i.e*., UV327, UV328 and AO246 (October 2025).^53^ The fact that we identified an additional 22 PBT substances is because 46% of the AOs compiled are not registered under REACH, which can partially be due to small production volumes of specific AOs and because even for registered AOs, persistence data are often lacking (Figure 1A, Appendix T2).

Our data set further makes it possible to identify benign AOs, *i.e.*, three of the investigated AOs are classified as fully non-hazardous (*i.e*., tert-amyl peroxy-2-ethylhexanoate, 2,4-diethenyl phenol, and octyl gallate). Another 16 AOs are classified as “only NRB”, which is not considered as a definite hazard end point in regulatory frameworks,^42^ so that those compounds are also regarded as non-hazardous in the following. Further, 49 AOs are classified as “only M” and 28 as NRB-M.

A comparison of hazards assigned to natural and synthetic AOs revealed that all PBT and PMT AOs are of synthetic origin. In contrast, 50% of the benign, “only M” and NRB-M AOs can be found naturally (). Studies focusing on hazard comparison of natural *vs* synthetic chemicals are limited, but literature on pesticides suggests that synthetic compounds are generally more persistent than natural compounds.^54^ Our data confirm this trend as only four out of the 137 AOs classified as P occur naturally. In terms of toxicity, differences between natural and synthetic compounds are less clear^54,55^ —in our case, 48% of the studied natural AOs are classified as T. While these findings suggest that natural AOs may be a safer alternative, it has to be noted that a full hazard and risk assessment requires to further consider production volumes, usage patterns, and emission pathways of natural and synthetic antioxidants.

### Structural Similarity of Hazardous and Non-hazardous AOs

To explore the relationship between the AOs’ molecular structure, functionality and hazard profile, we visualized structural similarities using the t-SNE dimensionality reduction method (Figure 1C). Although global distances in t-SNE embeddings are not directly interpretable and results depend on hyperparameter selection,^56^ the method is well suited for exploratory visualization and helps identifying several broad trends. Of the compounds classified as PBT, PMT, and PT, the majority are primary AOs and are located among phenolic (58%) and amine (10%) structures. In the group of secondary AOs, phosphor-containing structures are the most common hazardous AOs (14%, *i.e.*, 36% of all phosphor-containing AOs studied). In contrast, within the group of safer AOs (*i.e*., non-hazardous, “only M”, NRB-M), synthetic primary AOs are less present (i.e., 21% of synthetic phenols and amines), while natural primary AOs including carboxylic acids, structures with an aliphatic alcohol group, or secondary AOs dominate (Figure 1C, Figure S6).

Yet, seven of the 19 non-hazardous AOs are synthetic primary AOs, suggesting safer alternatives may be possible also for primary AOs. The categorization of these 19 AOs as non-toxic is partly backed by experimental data, while their non-persistent categorization relies fully on *in silico* data, which comes with limitations are elaborated previously. As BIOWIN generally predicts ester groups, aromatic alcohols and linear carbon chains affect mineralization positively, secondary AOs are often categorized as not P, and hence less hazardous, as they often contain ester groups, which can be readily hydrolyzed. The structural analysis further points out limitations in hazard classification schemes, which rely on threshold-based criteria.^57^ For instance, two sterically hindered phenols with very similar structures fall into different hazard classes—one is classified as benign and the other as PBT (structures shown in upper right corner of Figure 1C). This is due to slight differences in estimated values for persistence and bioaccumulation. The PBT-classified compound has a longer alkyl chain, resulting in a higher molecular weight and log *K*_ow_, parameters that lead to increased values in model predictions for P and B, respectively. Experimental data support the classification of the PBT compound as it has been shown to possess toxicity toward reproduction and specific target organ toxicity (Appendix T2), while no corresponding data are available for the structurally similar benign analogue. This example illustrates how small structural differences can lead to divergent classifications, underscoring the need for careful interpretation of threshold-based assessments.

To investigate whether electronic or physico-chemical properties of the AOs could further explain their hazard profiles, we examined relationships between hazard classification and HOMO–LUMO gap (as a proxy for chemical reactivity, calculated with g-xTB^58^) and log *K*_ow_ (as a measure of hydrophobicity and bioaccumulation potential, calculated following Wildman and Crippen^59^) as suggested by Kostal et al.^60^ In terms of toxicity, no significant separation of the toxic and nontoxic compounds was observed as a function of HOMO–LUMO gap and log *K*_ow_ (Figure S7). In contrast, a potential relationship appears for degradability: greater HOMO–LUMO gaps and lower log *K*_ow_ values associate with a higher likelihood of degradation (Figure S8). However, log *K*_ow_ and the HOMO–LUMO gap, in turn, are correlated positively and negatively, respectively, to the molecules’ complexity calculated following Bertz et al.^61^ The counterintuitive relation between a higher HOMO–LUMO gap (less reactive) and greater likelihood of degradation could therefore be explained by its spurious negative correlation with molecular complexity (less complex molecules are more biodegradable). The absence of a clear structure-toxicity relationship for AOs could potentially be explained by the fact that toxicity can be driven by reactive TPs in addition to the parent compound itself, and the heterogeneity of possible toxicity mechanisms. These effects would dilute global correlations and would be difficult to be picked up by descriptors of the parent AOs. Consequently, more diverse reactivity descriptors might need to be considered. Unfortunately, a fully comprehensive structure–hazard analysis is not feasible with the current data set due to limited sample size, heterogeneity of compound classes, and uncertainties in hazard estimates.

To enable more rigorous analysis in the future, several additions could strengthen the data set, *e.g.*, generating more experimental hazard data for AOs currently estimated non-hazardous. This would help balance the data set, also for machine learning, and improve confidence in identifying truly benign AOs. The AOs’ functionality must also be evaluated further based on their specific uses. For example, when used as rubber additives, phenolic AOs possess lower material antiaging efficiency compared to amine AOs. However, phenolic AOs have less effect on rubber color and thus are widely used in light-colored rubber products.^62^ Unfortunately, based on the data indicating an AO’s use cases publicly available in ECHA registration dossiers, it is not possible for us to further elucidate a link between use case and functionality of AOs.

### Formation of Toxic TPs

By design, most AOs act as reducing agents and form oxidized TPs. Yet, very few TPs have been identified for the wide range of chemicals used as AOs (Figure S9). One known TP is phosphate AO168=O (tris­(2,4-di-*tert*-butylphenyl)­phosphate), stemming from phosphite AO168 oxidation.^63^ No hazard data are available for AO168=O in the considered databases, though a previous study highlighted its contribution to cardiotoxicity in fish,^64^ as well as its environmental persistence and bioaccumulation potential.^22^

Diphenylamine AOs generally bear the potential to form intermediate quinone TPs when in contact with atmospheric ozone.^26,65^ For example, the common rubber additive P-phenylenediamine (PPD) AOs form PPD-quinones.^62,65^ In the case of 6PPD and IPPD, the quinone TPs exhibit greater toxicity toward aquatic life than the parent AOs.^66^ A famous synthetic phenolic AO is BHT. Five TPs of BHT have been identified, including one quinone TP, i.e., BHT–OH (2,6-di-*tert*-butyl-4-(hydroxymethyl)­phenol), BHT-CHO (3,5-di-*tert*-butyl-4-hydroxybenzaldehyde), BHT-COOH (3,5-di-*tert*-butyl-4-hydroxybenzoic acid), BHT-Q (2,6-di-*tert*-butyl-1,4-benzoquinone) and BHT-quinol (2,6-di-*tert*-butyl-4-hydroxy-4-methyl-2,5-cyclohexadienone).^67^ Based on our hazard assessment, only BHT-COOH can be considered non-toxic. Besides abiotic oxidation, laccase-mediated oxidative biotransformation of phenols is widely acknowledged.^68^ Further, the Brenda enzyme database^69^ suggests several monooxygenases (*e.g*., 4-nitrophenol 4-monooxygenase, 2,4,6-trichlorophenol monooxygenase, and pentachlorophenol monooxygenase) and 3-(hydroxyamino)­phenol mutase can transform phenols into quinone TPs, partially confirmed by studies in wastewater treatment systems.^70^ BHA, also a phenolic AO, is degraded to TBHQ (*tert*-butylhydroquinone), a hydroquinone which itself is also used as an AO.^71^ Further, TBHQ reacts to TQ (thymoquinone) in the presence of oxygen, metal ions, or hydroperoxides in oils and fats.^72^ Even though the mechanisms by which quinones cause toxic effects are complex, it has been shown that they create various adverse effects *in vivo*, including acute cytotoxicity, immunotoxicity, and carcinogenesis, highlighting concern over AO TPs.^73^

To complement our literature search on AO TPs, we investigated which hazards may occur when AOs enter the aquatic environment. Combining enviPath predictions^47^ of TP structures with ECOSAR^38^ toxicity predictions indicated that 186 out of 506 different predicted TPs may harm aquatic species (Appendix T4). They stem from 98 parent AOs, mostly amines and phenols. Of the 19 non-hazardous or “only NRB” AOs, only two synthetic amine AOs are predicted to form toxic TPs. This underscores the complexity of the problem and hidden risks from TPs. Examples of toxic TPs formed from non-toxic parents are discussed in more detail in SI 2.5. Still, toxicity assessments of predicted TPs bear great uncertainties and only a fraction of predicted TPs are actually found in the environment.^74^ Yet, it is evident that especially TPs of primary AOs can pose significant risk to human and environmental health, and future research should focus on identifying their structures and hazards. Alternatively, AOs are strong candidates to be studied with new approach methodologies (NAMs) using novel hazard end points such as the cumulative toxicity equivalents (CTE) and persistent toxicity equivalents (PTE). The CTE/PTE concept targets toxicity of parent compounds (i.e., before degradation, CTE) and the reaction mixture of parent compound and TPs after respective degradation assays (PTE), thereby allowing effect-based determination of toxic TPs by comparing CTE and PTE without the need to identify TP structures.^75^

## Supplementary Material





## Data Availability

List of AOs, all hazard data collected and script to reproduce Figure 1C are available from 10.25678/000FVT.

## Notes

Additional information on methods used for compiling AO list and to perform hazard assessment, as well as graphs supporting hazard assessment results (PDF)
List of AOs, AO transformation products, and full AO hazard assessment (XLSX)
