# Supplementary material for: Hazard Assessment of Antioxidants as Contaminants of Concern
Source: Environ Sci Technol Lett. 2026 Mar 2;13(4):560–7. doi: 10.1021/acs.estlett.5c01217 (PMC13085789; doi:10.1021/acs.estlett.5c01217)
Supplement: Supplementary file 1 [file ez5c01217_si_001.pdf]

Supporting Information

Hazard Assessment of Antioxidants as Contaminants of Concern

*Carolin Seller-Brison[a,b,#], Franziska Weissbach[c,#], Kjell Jorner[c], Martin Scheringer[d], Kathrin Fenner[a,b,]**

[a] University of Zurich, Department of Chemistry, Winterthurerstrasse 190, 8057 Zurich, Switzerland

[b] Eawag, Department of Environmental Chemistry, Überlandstrasse 133, 8600 Dübendorf, Switzerland

[c] ETH Zurich, Department of Chemistry and Applied Biosciences, Institute of Chemical and Bioengineering, Vladimir-Prelog-Weg, 1-5/10, 8093 Zurich, Switzerland

[d] ETH Zürich, Department of Environmental Systems Science, Universitätstrasse 16 8092 Zürich, Switzerland

*corresponding author: kathrin.fenner@eawag.ch

[#]joint first co-authors

Content

1. Supplementary Methods 3
1.1 Web of Science Search 3
1.2 Review of CAS numbers and SMILES strings 3
1.3 Hazard classification 3
2. Supplementary Results 5
2.1 Comparison of literature experimental data and *in silico* predictions 5
2.2 Data availability and hazards of antioxidants 6
2.3 Hazard comparison of natural and synthetic antioxidants 7
2.4 Structure analysis of hazardous and non-hazardous antioxidants 7
2.5 Antioxidant parent compounds and transformation products 9
References 12

List of Figures

Figure S1: Comparison of literature OECD 301 experimental data with model predictions 5
Figure S2: Comparison of literature experimental EC_{50} values for invertebrates with ECOSAR predictions 5
Figure S3: Comparison of literature experimental LC_{50} values for fish with ECOSAR predictions. 6
Figure S4: Data availability and hazards of antioxidants. 6
Figure S5: Hazard comparison of synthetic and natural antioxidants 7
Figure S6: Core structure of antioxidants and their assigned hazard profile. 7
Figure S7: HOMO-LUMO gap and $\log K_{ow}$ of antioxidants and their toxicity 8
Figure S8: HOMO-LUMO gap and $\log K_{ow}$ of antioxidants and their persistence 8
Figure S9: Structures of AO representatives and their TPs 10

List of Tables

Table 1: Criteria for hazard classification 3

1. Supplementary Methods

1.1 Web of Science Search

We systematically searched the Web of Science Core Collection database using the search phrase “synthetic phenolic antioxidants”. As we wanted to focus on antioxidants (AOs) most relevant for human and environmental exposure, we restricted the Web of Science Search to peer-reviewed research articles with further selection criteria “environmental science” and “environmental studies” to capture a significant literature body featuring environmental monitoring data. Articles with a publication date until October 24, 2024 were included. The compiled reference list contained 157 publications, which were then thoroughly reviewed for their titles and abstracts to identify whether specific chemical AO structures were reported in the respective study, which resulted in a list of 108 relevant articles. In those 108 articles, we screened which AOs were studied and whether AO names and CAS numbers were provided. Collected CAS numbers were compared to the already compiled AO list and complemented respectively. Besides synthetic phenolic AOs, several studies found through this literature search also reported not only phenolic AOs, but also AOs of different chemical structures, *e.g.*, phosphites, phosphates, or diphenylamines, which were included in our AO list as well. Even though our literature search was geared towards phenolic AOs, our overall list of AOs resulted in 186 phenols, 104 amines, 51 phosphor-based structures, and others, and is therewith a representative cross-section of structures having relevant uses as AOs.[1]

1.2 Review of CAS numbers and SMILES strings

The compiled list of AOs based on our literature search contained compound names and CAS numbers. We further curated this list by querying CAS numbers in SciFinder,[2] the CompTox Chemicals Dashboard,[3] PubChem[4] and the ECHA Chemicals Database.[5] Compounds with CAS numbers assigned to oligomers, compounds of unknown or variable composition, complex reaction products or biological materials (UVCBs), CAS numbers assigned to different structures when comparing across the four databases, or multiple CAS numbers assigned to only one structure were excluded from the substance list as this complicated the search for the substances hazard characteristics (relevant for eight AOs). SMILES strings were added to the dataset for all remaining structures.

1.3 Hazard classification

Table S1: Detailed criteria for hazard classification applied to curated list of antioxidants

Hazard	Hazard cut-off criteria
NRB	Recalcitrance to mineralization (not readily biodegradable (NRB)) was evaluated based on outcomes of OECD 301 studies. We employed cut-off criteria as given by the guideline, *i.e.*, a compound is considered NRB when there is less than 70% removal of dissolved organic carbon (DOC) and less than 60% of theoretical oxygen demand (ThOD) or theoretical CO_2 ($ThCO_2$) production for over 28 days of experiment. In case a compound shows more than 70% removal of DOC and

	more than 60% of ThOD or $ThCO_2$ production, these cut-off values have to be reached within a 10-day window of the overall 28-day experiment for the compound to be considered readily biodegradable (RB).[6]<br><br>In case no experimental data was available, predictions from BIOWIN6[7] and Körner et al.[8] were compared and considered for hazard assessment.[9] In case of conflicting model results, priority was given to predictions stemming from the model of Körner et al.[8]
P	Experimentally derived half-lives (DT_{50}) from OECD 308/309 simulation studies were used to evaluate persistence. An AO is considered P when it is NRB and its DT_{50} exceeds 40 days in freshwater and 120 days in sediment.[9,10]<br><br>In case an AO is NRB but no experimental DT_{50} data are available, Biowin 3 was used to predict P. In case the predicted ultimate biodegradation timeframe is >months (probability <2.2), the AO is considered P.[9]
T	Toxicity towards aquatic life was evaluated for fish and aquatic invertebrates. A compound was considered acutely toxic when the EC_{50} (48 hours) for aquatic invertebrates or the LC_{50} (4 days) for fish is <1 mg L^{-1}.[11] For chronic toxicity, the cut-off value for long-term no-observed effect concentration (NOEC) or EC_{10} for marine or freshwater organisms was set to <0.01 mg L^{-1}.[10,11] The same cut-off values were applied to ECOSAR[7] predictions in case experimental data was limited. In case no experimental data was available and ECOSAR[7] predictions did not yield reliable results for acute or chronic toxicity (*i.e.*, AOs with log K_{ow} >5), we considered the AOs predicted log K_{ow} values to estimate baseline toxicity. AOs with log K_{ow} >4 were considered T.[11]<br><br>For toxicity towards humans, we considered all compounds toxic that meet the criteria for classification as carcinogenic (C, category 1A or 1B), germ cell mutagenic (M, category 1A or 1B), toxic for reproduction (R, category 1A, 1B, or 2), or specific target organ toxicity after repeated exposure (STOT RE, category 1 or 2), as well as compounds that meet the criteria for classification as endocrine disruptor (ED).[10] In case this data was not available by the International Agency for Research on Cancer Classified Agents List,[12] or the Japanese GHS classifications list,[13] we further considered all compounds toxic that are labelled as CMR, ED or potentially ED in the ECHA database,[5] in safety data sheets provided by the chemical vendors (Appendix T5), or by Wiesinger et al.[14]<br><br>Further, we classified compounds as toxic when their safety data sheets suggest acute and chronic toxicity to aquatic life, liver toxicity, or neurotoxicity. Finally, in case none of the previous data could be found for a specific AO, we considered AOs with predicted Cramer Class III as toxic.[15]
B	We considered all AOs as bioaccumulating when their bioconcentration factor (BCF) in aquatic species is >2000.[10]
M	We used the more recently introduced mobility criterion to describe an AOs ability to travel long distances and to pass through natural or engineered system barriers. We classified an AO as mobile when its log K_{oc} is <3.[10]

2. Supplementary Results

2.1 Comparison of literature experimental data and *in silico* predictions

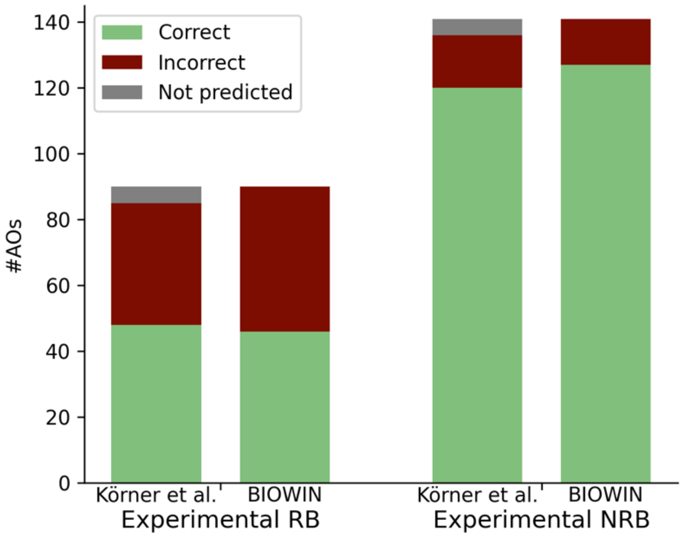

Figure S1: Comparison of literature OECD 301 experimental data (n= 231) with model predictions for ready biodegradability, e.g., by BIOWIN6[7] or the workflow outlined by Körner et al.[8] NRB = compounds that are not readily mineralized. RB = compounds that are readily mineralized. "Correct" stands for correctly predicted classification, "incorrect" for incorrectly predicted classification.

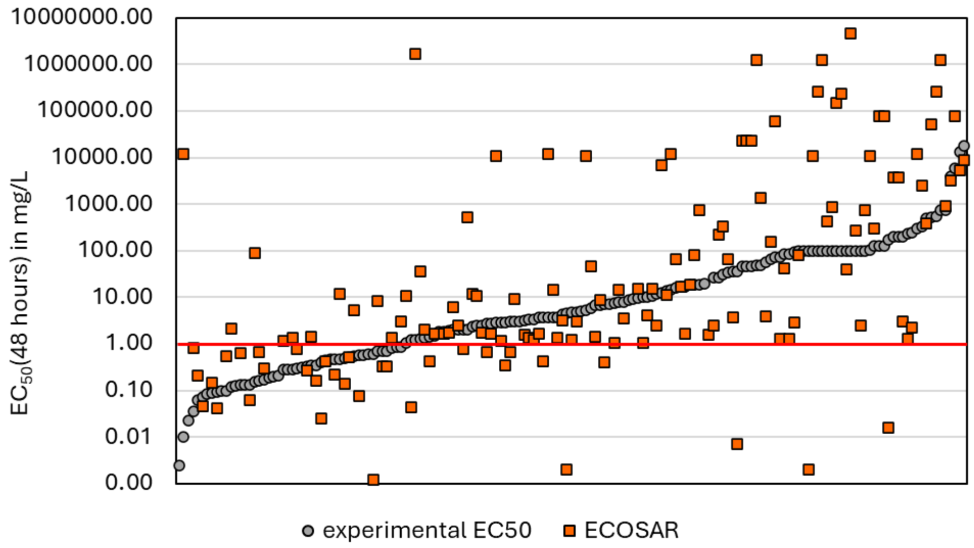

Figure S2: Comparison of literature experimental EC_{50} values (n=171) for invertebrates with ECOSAR[7] predictions. Red line indicates the cut-off criteria for acute toxicity, i.e., 1 mg/L.[11]

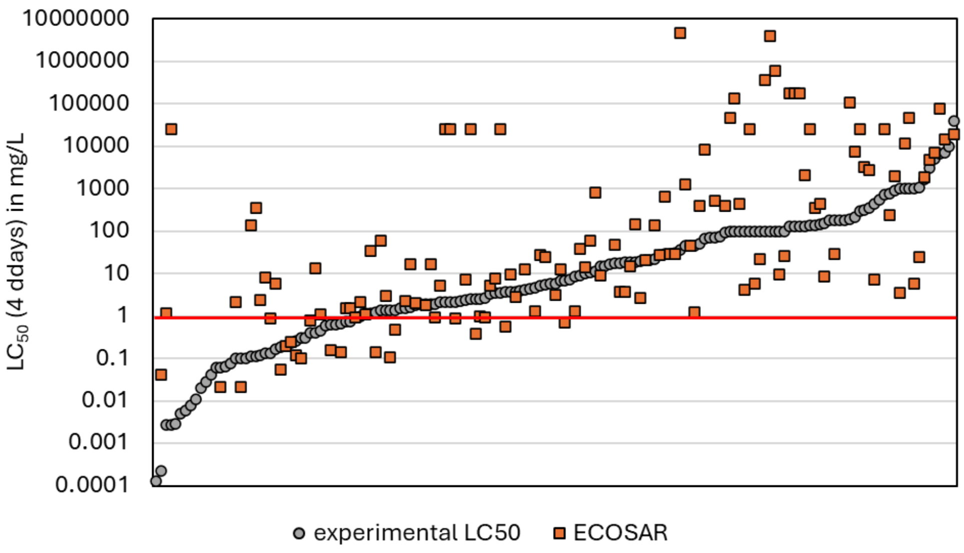

Figure S3: Comparison of literature experimental LC_{50} values for fish (n= 159) with ECOSAR[7] predictions. Red line indicates the cut-off criteria for acute toxicity, i.e., 1 mg/L. [11]

2.2 Data availability and hazards of antioxidants

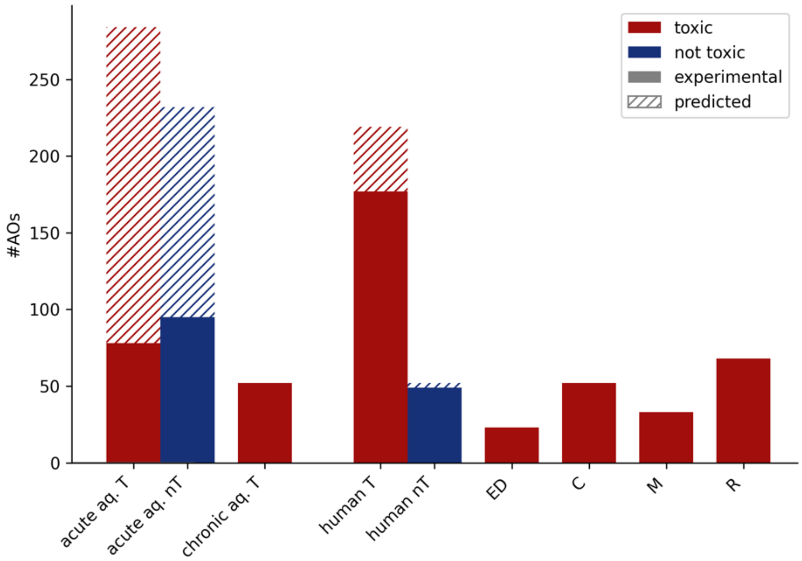

Figure S4: Detailed information on toxicity hazards identified for antioxidants (AOs). Solid bars show numbers of antioxidants with literature experimental data for the respective toxicity endpoint. Hatched bars show number of antioxidants for which the respective toxicity endpoint was predicted in silico.

2.3 Hazards comparison of natural and synthetic antioxidants

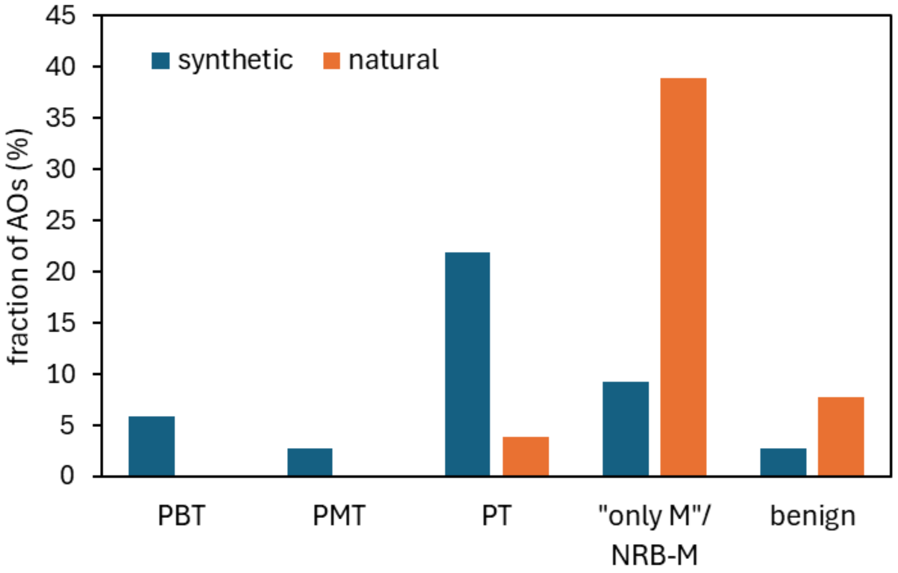

Figure S5: Hazard comparison of synthetic (n=413) and natural (n=103) antioxidants (AOs). The fractions shown are corrected for the respective number of AOs in each group, not total number of AOs studied.

2.4 Structure analysis of hazardous and non-hazardous antioxidants

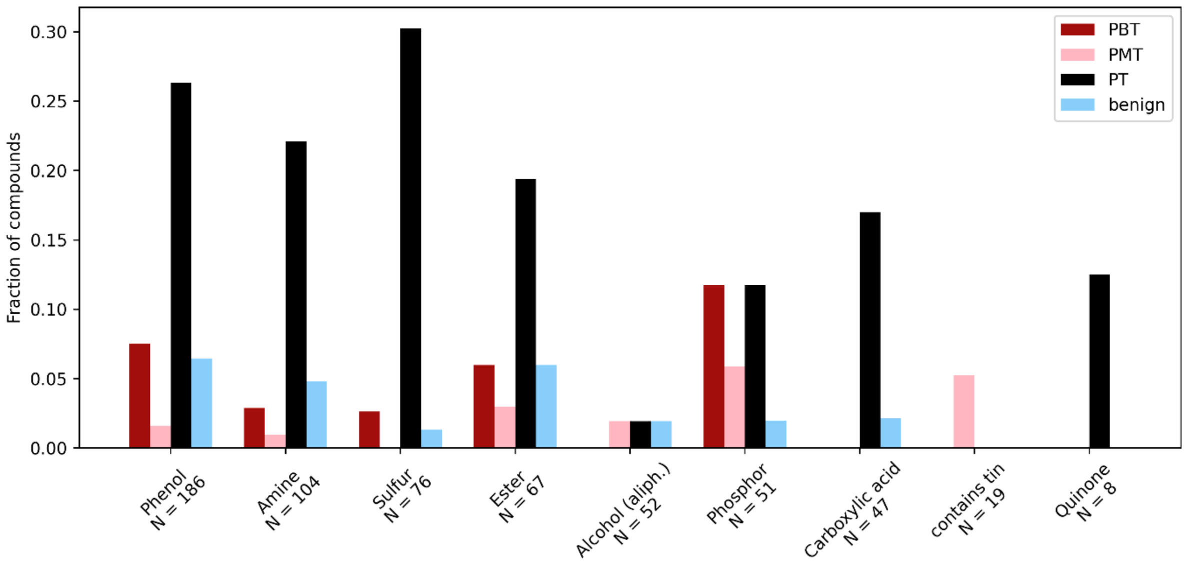

Figure S6: Antioxidants sorted according to presence of frequently found functional groups and the frequency of specific hazard profiles (i.e., PBT, PMT, PT, no hazard aka "benign") in each of these groups. Compounds can contain multiple functional groups. In that case, they count towards each of these groups.

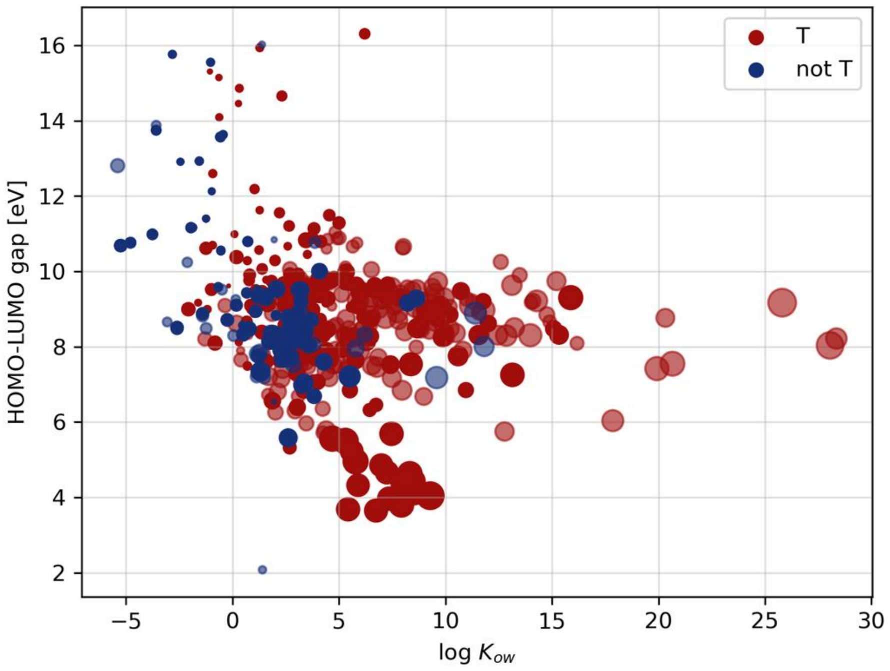

Figure S7: HOMO-LUMO gap and log K_{ow} of antioxidants and their toxicity towards aquatic organisms and humans. The size of the markers represents the complexity of the structures calculated via the BertzCT topological index.[16]

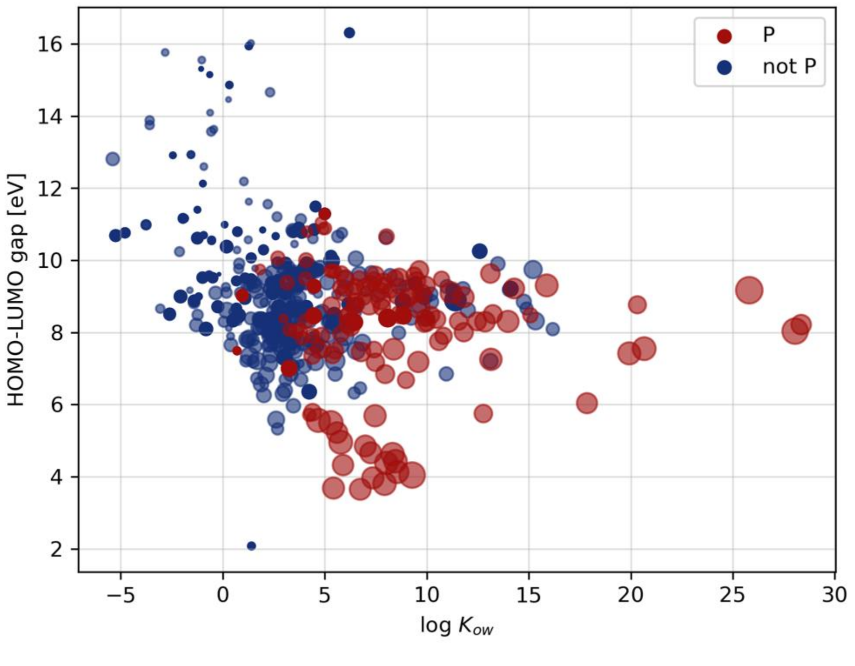

Figure S8: HOMO-LUMO gap and log K_{ow} of antioxidants and their persistence. The size of the markers represents the complexity of the structures calculated via the BertzCT topological index.[16]

2.5 Antioxidant parent compounds and transformation products

AO168 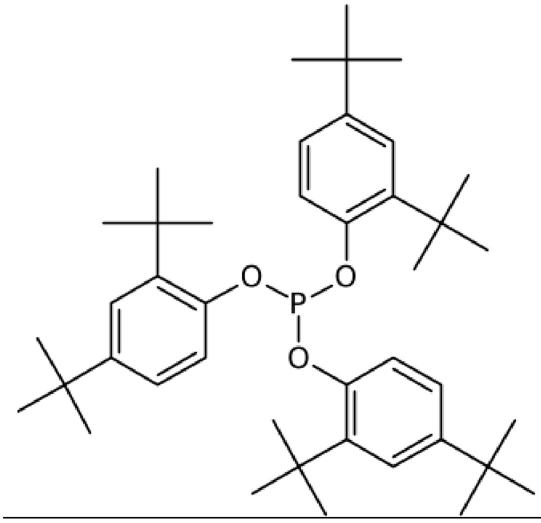	AO168=O 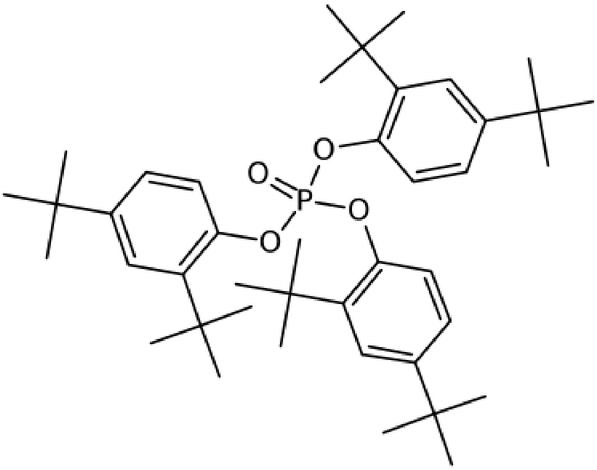
6PPD 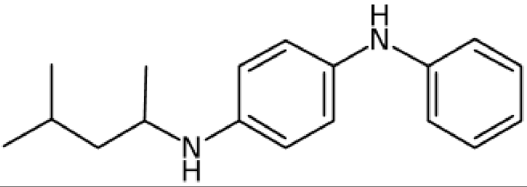	6PPD-Q 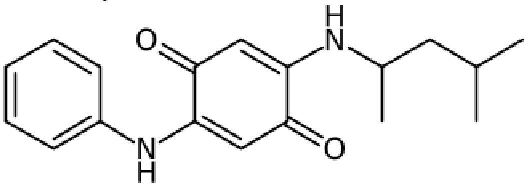
IPPD 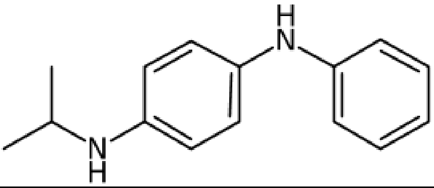	IPPD-Q 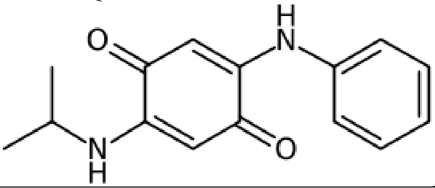
BHT 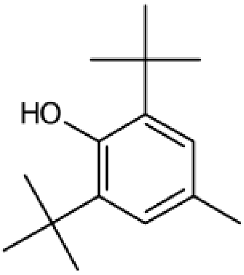	BHT-CHO 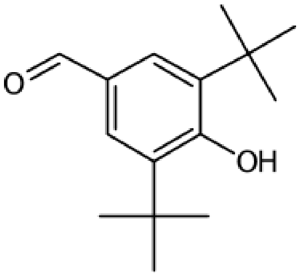<br><br>BHT-COOH

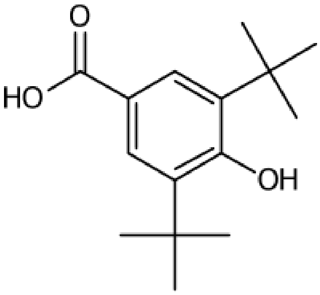

BHT-Q

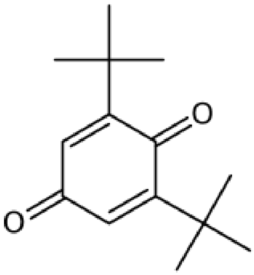

BHT-quinol

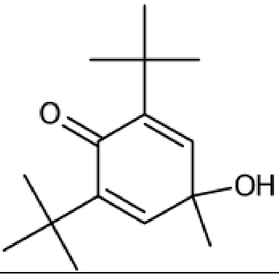

BHA

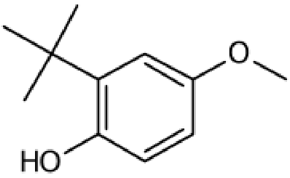

TBHQ

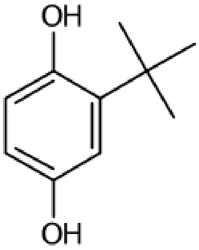

TQ

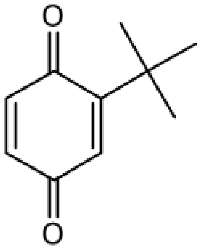

Figure S9: Structures of AO representatives and their transformation products (TPs). Parent compounds shown on the left, TPs on the right.

For 11 non-toxic parent compounds, predicted transformation products were assessed to be toxic to aquatic life. In the majority of these cases bisarylated secondary amines were predicted to be transformed into monoarylated primary amines. These primary aromatic amines are known to be toxic to aquatic life[17] and their excess toxicity is recognized by ECOSAR in the class of unhindered anilines. They are primarily predicted to be formed via enviPath rule bt0374, which describes 1,2-dioxygenation and cleavage of aromatic rings connected by an ether, amine or carbonyl group.

References

(1) Gulcin, İ. Antioxidants: A Comprehensive Review. *Arch. Toxicol.* **2025**, *99* (5), 1893–1997. https://doi.org/10.1007/s00204-025-03997-2.

(2) American Chemical Society (ACS). *CAS SciFinder*. https://scifinder-n.cas.org/ (accessed 2025-1022).

(3) US Environmental Protection Agency (EPA). *CompTox Chemicals Dashboard*. https://www.epa.gov/comptox-tools/comptox-chemicals-dashboard (accessed 2025-11-20).

(4) National Library of Medicine (NIH). *PubChem Explore Chemistry*. https://pubchem.ncbi.nlm.nih.gov/ (accessed 2026-02-16).

(5) European Chemicals Agency (ECHA). *ECHA substance information*. https://echa.europa.eu/information-on-chemicals (accessed 2025-11-20).

(6) OECD. *Test No. 301: Ready Biodegradability*; OECD, 1992. https://doi.org/10.1787/9789264070349-en (accessed 2025-11-20).

(7) US Environmental Protection Agency (EPA). EPI Suite - Estimation Programs Interface Suite™ for Microsoft® Windows. United States Environmental Protection Agency: Washington, DC 2012.

(8) Körner, P.; Glüge, J.; Glüge, S.; Scheringer, M. Critical Insights into Data Curation and Label Noise for Accurate Prediction of Aerobic Biodegradability of Organic Chemicals. *Environ. Sci. Process. Impacts* **2024**, *26* (10), 1780–1795. https://doi.org/10.1039/D4EM00431K.

(9) ecetoc. *Information to Be Considered in a Weight-of-Evidence-Based PBT/VPvB Assessment of Chemicals (Annex XIII of REACH) - Special Report No. 18*; Brussels, 2014. https://www.ecetoc.org/publication/special-report-18-information-to-be-considered-in-a-weight-of-evidence-based-pbt-vpvb-assessment-of-chemicals-annex-xiii-of-reach/ (accessed 2026-02-16).

(10) The European Commission. Commission Delegated Regulation (EU) 2023/707 of 19 December 2022 Amending Regulation (EC) No 1272/2008 as Regards Hazard Classes and Criteria for the Classification, Labelling and Packaging of Substances and Mixtures (Text with EEA Relevance). *Official Journal of the European Union* **2022**. https://eur-lex.europa.eu/eli/reg_del/2023/707/oj/eng (accessed 2026-02-16).

(11) EcoMole. *REACH Online - Annex 4.1.2.: Classification criteria for substances*. https://reachonline.eu/clp/en/annex-i-4-4.1-4.1.2.html (accessed 2025-11-20).

(12) World Health Organisation (WHO). *IARC Monographs on the Identification of Carcinogenic Hazards to Humans*. https://monographs.iarc.who.int/search (accessed 2025-11-20).

(13) National Institute of Technology and Evaluation (NITE). *GHS Classification Results*. https://www.chem-info.nite.go.jp/chem/english/ghs/ghs_download.html (accessed 2025-11-20).

(14) Wiesinger, H.; Wang, Z.; Hellweg, S. Deep Dive into Plastic Monomers, Additives, and Processing Aids. *Environ. Sci. Technol.* **2021**, *55* (13), 9339–9351. https://doi.org/10.1021/acs.est.1c00976.

(15) Patlewicz, G.; Jeliazkova, N.; Safford, R. J.; Worth, A. P.; Aleksiev, B. An Evaluation of the Implementation of the Cramer Classification Scheme in the Toxtree Software. *SAR QSAR Environ. Res.* **2008**, *19* (5–6), 495–524. https://doi.org/10.1080/10629360802083871.

(16) Bertz, S. H. The First General Index of Molecular Complexity. *J. Am. Chem. Soc.* **1981**, *103* (12), 3599–3601. https://doi.org/10.1021/ja00402a071.

(17) Gheni, S. A.; Ali, M. M.; Ta, G. C.; Harbin, H. J.; Awad, S. A. Toxicity, Hazards, and Safe Handling of Primary Aromatic Amines. *ACS Chemical Health & Safety* **2024**, *31* (1), 8–21. https://doi.org/10.1021/acs.chas.3c00073.
